# Intention to use digital mental health solutions: A cross-sectional survey of university students attitudes and perceptions toward online therapy, mental health apps, and chatbots

**DOI:** 10.1177/20552076231216559

**Published:** 2023-11-30

**Authors:** Elton Fayiah Gbollie, Jason Bantjes, Lucy Jarvis, Sonja Swandevelder, Jean du Plessis, Richard Shadwell, Charl Davids, Rone Gerber, Nuhaa Holland, Xanthe Hunt

**Affiliations:** 1 Department of Psychiatry, 121470 Faculty of Medicine and Health Sciences, 26697 Stellenbosch University, Stellenbosch, South Africa; 2 Mental Health, Alcohol, Substance Use and Tobacco Research Unit, SAMRC, South Africa; 3 Department of Psychiatry and Mental Health, 37716 University of Cape Town, Cape Town, South Africa; 4 71859 Western Cape Department of Health, Tygerberg Hospital, Cape Town, South Africa; 5 Biostatistics Unit, 59097 SAMRC, Cape Town, South Africa; 6 Institute for Life Course Health Research, Department of Global Health, 26697 Stellenbosch University, Stellenbosch, South Africa; 7 Center for Student Counselling and Development, 26697 Stellenbosch University, Stellenbosch, South Africa; 8 Student Development and Support, 56390 University of the Western Cape, Cape Town, South Africa

**Keywords:** mhealth, digital mental health, chatbot, online therapy, student mental health, youth mental health, ehealth

## Abstract

**Background:**

Globally, the high prevalence of mental disorders among university students is a growing public health problem, yet a small minority of students with mental health problems receive treatment. Digital mental health solutions could bridge treatment gaps and overcome many barriers students face accessing treatment. However, there is scant evidence, especially in South Africa (SA), relating to university students’ use of and intention to use digital mental health solutions or their attitudes towards these technologies. We aim to explore university 2students attitudes towards and perceptions of digital mental health solutions, and the factors associated with their intention to use them.

**Methods:**

University students from four SA universities (*n* = 17 838) completed an online survey to assess experience with, attitudes and perceptions of, and intentions to use, digital mental health solutions. We conducted an exploratory factor analysis to identify factors underlying attitudes and perceptions, and then used multivariate ordinal regression analysis was used to investigate the factors’ association with students’ intention to use digital mental health solutions.

**Results:**

Intention to use digital mental health solutions was high, and attitudes towards and perceptions of digital mental health solutions were largely positive. Importantly, our analysis also shows that 12.6% of users were willing to utilise some form of digital mental health solutions but were unwilling to utilise traditional face-to-face therapies. The greatest proportion of variance was explained by the factor ‘Attitudes towards digital technologies’ utility to improve student counselling services, provided they are safe’.

**Conclusion:**

SA university students are already engaging with digital mental health solutions, and their intention to do so is high. Certain attitudes and perceptions, particularly concerning the utility, effectiveness, and safety, underlie willingness to engage with these solutions, providing potential targets for interventions to increase uptake.

## Background

The prevalence of undiagnosed and untreated mental disorders among university students is a growing public health problem globally^1^ and in South Africa (SA),^2^ about 20.3% of students meet the diagnostic criteria for one or more mental disorders.^3^ In SA, the lifetime prevalence (38.5%)^4^ and 12-month (31.5%)^4^ and 30-day (53.3%)^2^ prevalence for any common mental disorder, is higher than the country's general population. The 30-day prevalence of suicidal ideation among students in the country is 24.4%.^5^ Two of the most commonly reported mental disorders amongst university students, mood and anxiety disorders, have been linked to adverse social and academic outcomes^6^ and increased risk of suicide.^7^ Despite high prevalence, most students with mental disorders do not access treatment.^8^ In SA, only between 21.3%^9^ and 28.9%^10^ of students with mental health problems receive treatment.^11^ There are many reasons why students with mental health problems do not access treatment, including lack of perceived need, perceived ineffectiveness of treatment, issues with access to care and perceived inconvenience. Additionally, university students, as a group do not access treatment because of concerns about stigma, negative views of treatment, shame related to experiencing mental disorders, scepticism about treatment efficacy, mistrust of providers, busy schedules, normalising mental disorders, preferring to manage mental health problems on their own, high costs associated with accessing treatment, perception that staff in campus mental health centres are unfriendly, and long wait times for services.^10,12–16^

In an effort to reduce the significant treatment gap, universities have attempted to increase the scope and scale of student support and mental health services.^17^ However, the number of students in need of treatment greatly exceeds the resources of most counselling centres and institutions.^18^ Globally, many student counselling centres are underfunded and face staff shortages and burnout.^19^ The situation is even worse in low- and middle-income countries (LMIC), where student counselling services are frequently non-existent.^11^ There is a need to consider other scalable, accessible, and acceptable novel interventions to promote the mental health of vulnerable students, especially in LMICs such as SA, where resources for mental health care are severely constrained.

Digital mental health solutions, including online mental health resources, online therapy, mental health smartphone apps (apps), and mental health chatbots, could bridge treatment gaps, reduce patient waiting times and deliver interventions at lower costs.^20^ A growing body of evidence supports the use of technology to deliver mental health interventions for CMDs among students,^21^ with meta-analytic evidence showing that digital interventions have positive effects on depression and anxiety.^22^ Possible benefits of digital mental health solutions include increased convenience for the user regarding to time and location of treatment, the anonymity of services and avoidance of stigma, accessibility of such interventions, reduced costs for healthcare providers and the ability to bridge gaps in the provision of care.^20,23,24^ Understanding students’ willingness to use these technologies and their attitudes towards them is integral to planning student-centre mental health services. University students in high income countries (HICs) have expressed an interest in and intention to engage with digital mental health services, and have advocated for digital health services as a feasible technique for enhancing mental health care on campus.^25,26^ For instance, in a US study, 20–25% of students were interested in at-cost digital mental health solutions and 70–72% were interested in free digital mental health solutions.^26^ Yet, while there is some preliminary evidence to suggest that some SA university students find digital mental health solutions acceptable and helpful, there is little research from SA exploring student's utilisation of these digital resources and the factors associated with their intention to use digital solutions to promote their mental health.^27,28^

In the former respect, research studies have shown that a range of digital interventions might be acceptable and feasible to deliver among SA university students, but some limitations exist. For instance, researchers found that a brief semi-guided internet-based cognitive behavioural therapy (iCBT) for depression appealed to university students because of perceived anonymity, privacy and accessibility, but experienced disappointment at the lack of human contact.^28^ A more recent qualitative study by Hunt, Jivan^29^ showed that university students who were enrolled in a trial of online group cognitive behavioural therapy (GCBT) enjoyed some aspects of the groups’ digital delivery, but noted marked weaknesses of it too, again pointing to the lack of in-person human contact as a weakness.

The present study set out to expand what is known with a view to informing practice in the provision of student mental health services in SA and other low-resource settings by: (1) describing SA university students’ past utilisation of digital mental health solutions (i.e., online therapy, internet searches for mental health information, mental health applications (apps), and mental health AI Chatbots), as well as their intention to use these digital solutions ; (2) exploring students’ attitudes towards and perceptions of these digital mental health solutions; and (3) identify factors (i.e., sociodemographic characteristics, attitudes, perceptions) associated with students’ intention to use digital mental health solutions.

## Methods

This cross-sectional online survey research was conducted with South African university students from three universities between 1 November 2022 and 16 December 2022.

### Respondents and procedure

A self-selected sample of undergraduate and post-graduate students from four SA universities were recruited via email to complete an anonymous online self-report survey administered using Redcap. The universities (two from the Western Cape, one from Gauteng, and one national) were selected to achieve wide geographic reach and diversity in student demographics. The email invitation was distributed by participating institutions and the study was conducted in English, given that this is the language of tuition at the four participating SA universities. In addition, three email reminders were sent out, a week apart, to remind students of the survey invitation. To participate in the survey, respondents needed to: (1) be a registered undergraduate or postgraduate student at one of the participating universities; (2) be aged 18 years or older; and (3) provide informed consent.

The Health Research Ethics Committee (HREC) of Stellenbosch University (N22/08/100) approved this study and institutional permissions were obtained from participating universities. The research was conducted in accordance with the Declaration of Helsinki^30^ and the Guidelines for Good Clinical Practice.^31^ As an incentive to encourage participation, students were offered the opportunity to win one of five R500 (approximately $30) gift vouchers. Informed consent was obtained electronically prior to data collection. Data were de-identified and stored in a password protected cloud-based server.

### Measures

Respondents completed an anonymous self-report online survey which was compiled by the authors (see Supplementary materials S1) using survey items from a range of instruments used in similar studies to explore the use of digital technologies for healthcare,^32–39^ as well as items informed by the *Andersen Behavioural Model of Health Service Utilization,*^40^ which has been used as a conceptual framework to identify variables which predict health care service utilisation (including predisposing factors, enabling factors, and need).

The following data were collected:

1. Demographic information and digital technology use: Respondents were asked about their self-identified gender, population group, whether they were undergraduate or post graduate students, whether they owned a smart phone, how confident they felt using digital technologies, their social media use, their mains of accessing the internet, and their average monthly spend on internet access.
2. Previous treatment seeking: Respondents were asked to provide a subjective assessment of their current mental health status, their functioning, and their past, current, and 12-month engagement with psychological counselling or psychotherapy, or use of medication, for an emotional/psychological or substance use problem.
3. Experience using digital technologies and digital mental health solutions: Respondents were asked about past use of mental health apps, online video conferencing to consult with a mental health professional, and mental health chatbots. This included questions about frequency and, duration of use, perceived utility, and how respondents had learned about the digital solution.
4. Intention to use traditional therapy and digital mental health solutions: Respondents were asked a series of four-point Likert scale items about their intention to make use of traditional (i.e., face to face psychotherapy) and digital mental health solutions (i.e., internet searches for mental health resources, online therapy via videoconferencing, mental health apps, and mental health chatbots). Response options were given on a five point Likert scale where 1  =  ‘not at all likely’ and 5  =  very likely’.
5. Attitudes towards and perceptions of digital technology: Respondents were presented with a series of statements about technology and asked to indicate their level of agreement. These statements assessed respondents’ perceptions about whether digital mental health solutions could support the delivery of accessible, effective, affordable, convenient, private and anonymous, and user-friendly student counselling services (e.g., ‘Digital technologies could help to deliver more effective student counselling services’). We also assessed respondents’ attitudes towards Artificial Intelligence (AI) (e.g., ‘If I had to have a blood test, I would prefer to have the blood drawn by AI robot, rather than a nurse/doctor’), and privacy online (e.g., ‘I am concerned about data security and privacy when I use apps and other digital technologies’). Response options were given on a five point Likert scale where 1  =  ‘strongly disagree’ and 5  =  ‘strongly agree’.
6. Attitudes towards and perceptions of mental health apps: Respondents were asked to indicate how important (on a four-point Likert scale) the relative importance of various characteristics of digital mental health solutions, including data protection and privacy (e.g., Data protection and privacy) and local development (e.g., Apps developed by South Africans). Response options were given on a four point Likert scale where 1  =  ‘not at all important’ and 5  =  ‘very important’.
7. Attitudes towards and perceptions of online therapy: Respondents were asked a series of four-point Likert scale items about their attitudes towards and perceptions of online therapy. These items concerned whether respondents perceived online therapy to be convenient (e.g., ‘Online therapy is more convenient than face-to-face therapy’), private (e.g., ‘Online therapies are more private because you don’t have to go to the counselling Centre to get help’), and effective (e.g., ‘Online therapy programs are as effective as conventional face-to-face psychotherapies’). Response options were given on a four point Likert scale where 1  =  ‘not at all important’ and 5  =  ‘very important’.
8. Attitudes towards and perceptions of AI: Respondents were asked a series of questions about past use of chatbots, including frequency of use, duration of use, and level of comfort talking to chatbots about their mental health.

### Data analysis

Data were checked and cleaned before being imported into R and Statistica for statistical analysis and data visualisation. Descriptive statistics were used to describe sample characteristics, past and current use of various digital mental health solutions, intention to use digital mental health solutions, and attitudes towards and perceptions of using these technologies. We also produced an Upset plot for the 5 × 5 combinations of willingness to use various mental health solutions using the ComplexUpset package for R.^32^ We conducted an exploratory factor analysis, using an oblique rotation and only including variables with loadings of >0.5, to investigate if there were common factors underlying the various attitudes and perceptions about digital technologies and mental health solutions. Multivariate ordinal regression analysis was then used to investigate factors associated with students’ intention to use digital mental health solutions. In these final regression models we included sociodemographic variables and intention to use traditional therapies as co-variates so that we could control for the effect of these on intentions to use digital solutions. The eta-coefficients in the ordinal regression models were exponentiated to yield odds ratios (ORs).Results of all regression analyses are reported as odds ratios (ORs) with 95% confidence intervals (95% CI). For all tests of statistical analysis, the alpha was set to 0.05.

## Results

### Sample characteristics

The sample characteristics are summarised in Table 1.

**Table 1.** Sample characteristics (*n* = 17,838).

**Socio-demographic characteristics of the sample**		
Gender	Male	27,5%
	Female	71,8%
	Gender non-conforming	0,7%
Population group	Black-African	82,5%
	Coloured	5,3%
	Asian or Indian	3,0%
	white	7,7%
	Other (including prefer not to say)	1,5%
Student status	Undergraduate students	84,2%
	Postgraduate students	15,8%
**Mental health status and history of receiving treatment for a mental health problem**		
Self-reported current mental health status	Excellent	31,6%
	Good	36,1%
	Fair	24,3%
	Poor	8,0%
Among students with poor mental health, how difficult is it to do work, take care of things at home, or get along with other people.	Not difficult at all	1,2%
	Somewhat difficult	26,0%
	Very difficult	42,1%
	Extremely difficult	30,7%
Proportion of students who report ever receiving *psychological counselling* for an emotional/psychological or substance use problem.		18,8%
Proportion of students who report ever receiving *medication* for an emotional/psychological or substance use problem.		11,2%
Proportion of students who report receiving any treatment (counselling or medication) *in the last 12 months*		8,7%
**Access to technology and confidence using technology**		
Own a smart phone		97,6%
Confidence to use computers, smartphones and the internet	Not confident	1,3%
	Somewhat confident	6,2%
	Fairly confident	31,1%
	Very confident	61,4%
Internet access	Mobile data (using my cell phone)	75,7%
	Wi-Fi Fibre connection at home	33,1%
	Wi-Fi hotspots in public places	19,9%
	On campus	16,7%
	Other form of access	2,1%
	No internet access	
Average amount of money spend on data each month	Have unlimited (uncapped) free data paid for by my parents	9,9%
	Spend more than ZAR500/USD20 a month on data	15,4%
	Spend between ZAR200/USD10 and ZAR500/USD20 on data	30,0%
	Spend ZAR100/USD5 to ZAR199/USD10 a month on data	26,6%
	Spend less than ZAR100/USD5 on data	18,1%
**Social media use**		
Social media use (frequency of use on average)	I don't ever access social media	1,6%
	A few times a week	10,8%
	About once a day	7,6%
	Two or three times a day	26,1%
	More than three times a day	53,9%

#### Demographic characteristics

The survey received 17 838 responses. The majority of respondents identified as female (71.8%), Black African (82.5%), and undergraduate students (84,2%).

#### Mental health status and treatment history

92% of the sample reported that their mental health was excellent, good or fair. It is notable that, among the 8% of respondents who reported poor mental health, the impairments in functioning associated with poor mental health were great, with over half reporting that it was very difficult (42.1%) or extremely difficult (30.7%) to work, take care of things at home, or get along with other people. It can be noted that only 18.8% of all students had ever received psychological counselling for an emotional/psychological or substance use problem and 11.2% had ever received medication for an emotional/psychological or substance use problem. In addition, 8.7% reported receiving any treatment (counselling or medication) in the prior 12 months.

#### Access to technology and confidence using technology

The vast majority (97.6%) reported owning a smartphone and nearly two-thirds (61.4%) reported high confidence levels in using computers, smartphones, and the internet. The most common source of access to the internet was mobile data on a cell phone (75.7%), but Wi-Fi or Fibre connection at home (33.1%) or in public (19.9%) were also commonly reported. Over half of the sample spent between ZAR100-ZAR199/USD5-USD10 (30.0%) or ZAR200-ZAR500/USD10-USD25 (26.6%) a month on data.

#### Social media use

Over half of the sample (53.9%) reported using social media more than three times a day. A Spearman's rank correlation coefficient (ρ) for frequency of social media use (average hours per week) and mental health status found a significant moderate correlation, with poorer mental health associated with more frequent social media use (ρ = 0.048, 95% CI = 0.032 to 0.064), *p* < .001).

### Experiences of using digital mental health solutions and intention to use these technologies in the future

Table 2 summarises respondents’ experiences using digital mental health solutions and their intention to use them in the future. Only 12.4% of respondents reported ever having downloaded or used an app to help with mental health or emotional wellbeing. Among those who downloaded apps, 12.4% primarily used apps for stress (6.4%), anxiety (6.3%), mindfulness training (4.4%) and depression (4.3%), followed by addressing sleep problems (3.6%), improving coping skills (2.5%), and tracking emotions (2.1%). Among those who had downloaded or used apps in this way, more than half reported using the app only once or twice (32.3%), or 3–5 times (29.2%). However, 19.5% reported using the app over 20 times, with much lower proportions using it between 6 and 20 times, suggesting that most students either do not frequently engage with apps once downloaded or engage very frequently. A similar pattern was observed in respondents’ reports of the total duration of their use of a mental health app, which ranged from less than a week (22.5%), to more than a week but less than a month (25.6%), to more than a month but less than 3 months (21.4%), to more than 3 months (30.4%). Respondents mostly learned about mental health apps through internet searches (42.4%), and only a small minority reported that they had not found the app of any utility (13.4%).

**Table 2.** Experiences of using digital mental health solutions and intention to use these technologies in the future.

Consulting a mental health professional online		
Ever consulted a mental health professional using an online video conferencing platform		9,2%
Among students who consulted a mental health professional online, how helpful did they find it?	Not at all helpful	10,3%
	Somewhat helpful	36,2%
	Helpful	30,2%
	Very helpful	23,3%
Experience of using mental health applications (apps)		
Proportion of students who have ever downloaded or used an app to help with mental health or emotional wellbeing		12,4%
Among students who downloaded an app, what was the app for:		
Stress		6,4%
Anxiety		6,3%
Mindfulness training		4,4%
Depression		4,3%
Sleep problems		3,6%
Coping skills		2,5%
Track emotions / mood monitoring		2,1%
Procrastination		1,9%
Traumatic event/experience		1,6%
Symptom tracking		0,8%
Meditation		0,3%
Medication monitoring		0,3%
Smoking		0,2%
Substance use		0,2%
Other		0,6%
Among those who downloaded an app, self-reported frequency of use	Only once or twice	32,3%
	3–5 times	29,2%
	6–10 times	12,6%
	11–20 times	6,4%
	More than 20 times	19,5%
Among those who downloaded an app, self-reported length of time the app was used	Less than a week	22,5%
	Less than a month	25,6%
	Less than 3 months	21,4%
	More than 3 months	30,4%
Among students who used an app, where did you learn about the app	Internet search	42,4%
	Social media/advertising	33,4%
	Suggested by a friend	11,1%
	Suggested by a mental health professional	7,4%
	Other	5,8%
Among students who downloaded an app, how useful was the app?	Not at all helpful	13,4%
	Somewhat helpful	41,6%
	Helpful	25,0%
	Very helpful	20,0%
Made use of mental health chatbots		
Made use of a mental health chatbot		4,5%
Among students who used chatbots, self-reported frequency if use	Only once or twice	58,3%
	3–5 times	24,4%
	6–10 times	8,3%
	11–20 times	3,5%
	More than 20 times	5,5%
Among students who used a chatbot, self-reported length of use	Less than a week	54,8%
	Less than a month	22,3%
	Less than 6 months	16,6%
	More than 6 months	6,3%
Among students who used chatbots, where did they learn about them?	Internet search	50,8%
	Social media/advertising	30,2%
	Suggested by a friend	9,1%
	Suggested by a mental health professional	6,4%
	Other	3,5%
Among students who used a chatbot, how useful did they find it?	Not at all helpful	20,4%
	Somewhat helpful	39,3%
	Helpful	26,0%
	Very helpful	14,3%
Intention to use digital mental health solutions		
Traditional face-to-face mental health assistance (i.e., face-to-face therapy with a mental health professional)	Not at all likely	17,4%
	Somewhat unlikely	7,5%
	Somewhat likely	27,0%
	Very likely	48,2%
Online therapy with a real person using video conferencing	Not at all likely	25,0%
	Somewhat unlikely	14,6%
	Somewhat likely	31,0%
	Very likely	29,4%
Use the internet to look for mental health advice and/or resources	Not at all likely	18,9%
	Somewhat unlikely	12,6%
	Somewhat likely	30,3%
	Very likely	38,2%
Mental health apps	Not at all likely	28,1%
	Somewhat unlikely	18,4%
	Somewhat likely	31,7%
	Very likely	21,9%
Therapy with an AI (artificial intelligence) chatbot	Not at all likely	45,7%
	Somewhat unlikely	20,1%
	Somewhat likely	20,7%
	Very likely	13,5%

In addition, a total of 9.2% reported ever consulting a mental health professional online using video conferencing, of which the majority (89.7%) reported that online therapy was helpful.

By far the least utilised digital mental health solution were mental health chatbots, with 4.5% having utilised a chatbot for their mental health. Unlike with apps, where there were high rates of use at both ends of the spectrum (low and high frequency and duration of use), chatbot use tended to be low, with those reporting using chatbots mostly using them only once or twice (58.3%), or 3–5 times (24.4%). Similarly, most respondents used the chatbot for less than a week (54.8%) or less than a month (22.3%). While many respondents who had used chatbots found them helpful, a fifth (20.4%) reported them to be not at all helpful.

Respondents were, in the main, open to using digital mental health solutions in the future if they encountered a psychological or emotional problem, with 60.4% either very or somewhat likely to use online therapy with a real person using videoconferencing, 68.5% very or somewhat likely to use the internet to find mental health resources, 53.6% very or somewhat likely to use a mental health app, and 34.2% very or somewhat likely to use a mental health chatbot. Notably, between 20 and 25% of respondents said they were ‘not at all likely’ to use the various digital solutions (25.0% for online therapy, 18.9% for internet searches of mental health resources, 28.1% for apps, and 18.9% for chatbots). Moreover, the intention to use any type of digital mental health solution was lower than the intention to use traditional face-to-face modes of therapy where all but 17.4% intended to use.

**Figure 1.**
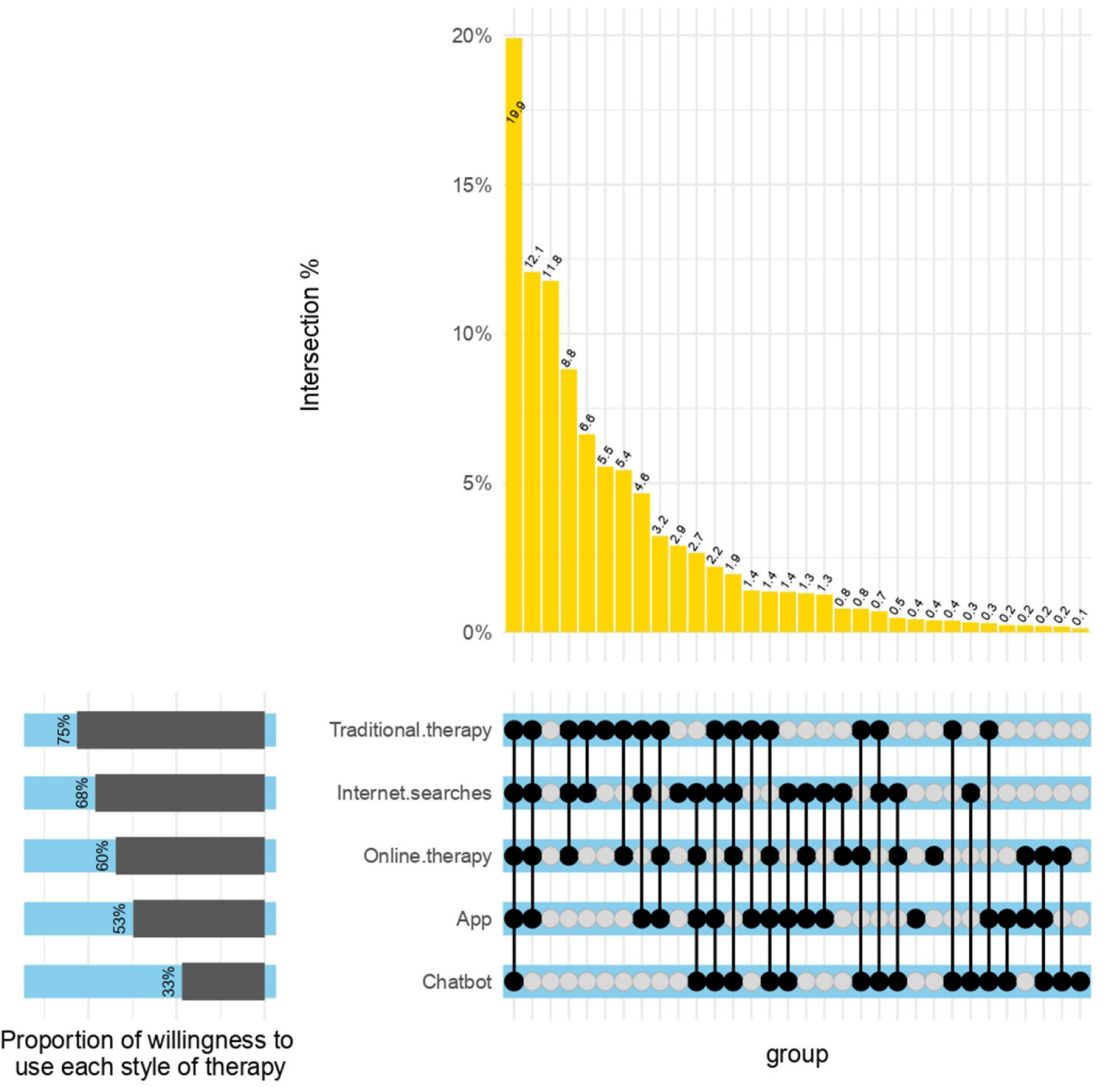
UpSet plot visualising intersections between intention to use digital and traditional mental health solutions. This UpSet Plot was rendered on R using ComplexUpset.^32^

The 5 × 5 combinations of respondents’ willingness to use the various digital solutions are graphically represented in an Upset Plot (see Figure 1). As can be seen in Figure 1, 19.9% of respondents’ intention to use all types of mental health solutions, digital or traditional. 11.8% did not intend to use any types of mental health solutions. As shown in the UpSet Plot, a small proportion (2.2%) of respondents intended to use only digital interventions, but not traditional face-to-face therapy. Furthermore, very small proportions of respondents only intended to use specific digital interventions, for instance, the 0.4% who only reported an intention to using apps (and no other solutions). Supplementary materials presents a breakdown of the (i) ‘mental health status’ and (ii) ‘history of receiving treatment for a mental health problem’ for participants who were willing and unwilling to use digital mental health solutions.

### Attitudes towards and perceptions of digital technology and digital mental health solutions

Table 3 summarises the data regarding attitudes towards and perceptions of digital technology and digital mental health solutions. Respondents endorsed that digital technologies could help deliver more accessible student counselling services (90%). However, they mostly disagreed with the idea that digital technologies could make counselling services more effective (69.3%) affordable (62.2%), convenient (65.3%), or private and anonymous (60.9%). The majority also felt that using digital technologies to provide mental health services must be controlled by legislation and regulations to protect users (90.5%) and expressed concern about data security and privacy when using apps and other digital technologies (84.7%). Data protection and privacy (84.7%), recommendation by a mental health professional (72.1%), and knowing that an app was approved by the health professions council or the government department of health (75.8%) were all thought to be very important by respondents, as was that apps be developed specifically for students (51.6%). Less than half the sample thought that recommendations by peers (46%) and local app development (44.6%) were very important, although the highest proportion of responses for these questions was still ‘very important’.

**Table 3.** Attitudes towards and perceptions of digital technology and digital mental health solutions.

	Strongly Disagree	Disagree	Agree	Strongly Agree
Digital technologies could help to deliver more **accessible** student counselling services	3,4%	6,7%	59,1%	30,9%
Digital technologies could help to deliver more **effective** student counselling services	13,7%	55,6%	4,2%	26,5%
Digital technologies could help to deliver more **affordable** student counselling services	8,6%	53,6%	3,5%	34,3%
Digital technologies could help to deliver more **convenient** student counselling services	9,2%	56,1%	3,2%	31,5%
Digital technologies could help to deliver more **private and more anonymous** student counselling services	12,5%	48,4%	4,3%	34,8%
Digital technologies could help to deliver more **user-friendly** student counselling services	11,9%	28,7%	3,6%	55,8%
The use of digital technologies to provide mental health services needs to be controlled by **legislation and regulations** to protect users	6,6%	2,9%	42,6%	47,9%
I am concerned about **data security and privacy** when I use apps and other digital technologies.	3,7%	11,5%	43,1%	41,6%
I believe superhuman **AI** (i.e., artificial intelligence that is even smarter than people) will be developed within the next 1 years	39,7%	13,8%	13,9%	32,7%
If I had to have a blood test, I would prefer to have the blood drawn by an **AI robot**, rather than a nurse/doctor	8,9%	15,7%	28,1%	47,2%
**Attitudes and perceptions about *mental health apps***				
If you were to try to choose a mental health app, how important would each of the following criteria be?				
	Not at all important	Not very important	Somewhat important	Very important
Data protection and privacy	1,5%	2,2%	11,6%	84,7%
Recommendations of other students	3,4%	10,7%	39,9%	46,0%
Recommendation by a mental health professional	1,4%	3,0%	23,5%	72,1%
Apps developed specifically for students	3,4%	10,3%	34,6%	51,6%
Apps developed by South Africans	7,1%	16,6%	31,7%	44,6%
Knowing that the app was approved by the health professions council or the government department of health?	1,9%	3,6%	18,7%	75,8%
**Attitudes and perceptions about *online therapy***				
	Strongly Disagree	Disagree	Agree	Strongly Agree
Online therapy will eventually replace face-to-face therapy	10,7%	28,3%	38,7%	22,3%
Online therapy is more convenient than face-to-face therapy	10,1%	28,8%	41,2%	19,9%
Online therapies are more private because you don’t have to go to the counselling centre to get help	6,9%	21,6%	43,6%	27,9%
Online therapy programs are less effective than conventional face-to-face therapy.	7,6%	35,5%	38,7%	18,2%
Trust in a therapist can be just as easily built on the Internet as in conventional face-to-face psychotherapy	12,2%	32,7%	37,9%	17,2%

We also included items which concerned perceptions of AI's capability relative to humans’. An inverse curve was observed in response to the item ‘I believe that AI which is even smarter than people will be developed in the next 1 year’, with 39.7% of respondents strongly disagreeing, and 32.7% strongly agreeing. However, for the item ‘If I had to have a blood test, I would prefer to have the blood drawn by an AI robot, rather than a nurse/doctor, the majority (73.3%) of respondents agreed or strongly agreed.

More than half of the respondents thought online therapy would eventually replace face-to-face therapy (61%) and felt that online therapy is more convenient than face-to-face therapy (61.1%). There was also a widespread endorsement of the benefits of online therapy from a privacy perspective (71.5%). However, the majority of respondents also perceived online therapy to be less effective than conventional face-to-face therapy (56.9%), and over a third of respondents did not think that a trusting therapeutic relationship could be built as well online as in person (44.9%).

### Factor structure underlying attitudes towards and perceptions of digital technologies for mental health

Our exploratory factor analysis of the variables assessing attitudes and perceptions of digital mental health technologies indicated that a 3-factor solution best fit the data. The three factors identified were: solution which identified the

1. Factor 1: Attitudes towards digital technologies’ utility to improve student counselling services, provided they are safe
2. Factor 2: Attitudes towards the effectiveness of online therapies, and trust in AI
3. Factor 3: Perception of the importance of data protection and human endorsement

Factor 1 (Attitudes towards digital technologies’ utility to improve student counselling services, provided they are safe) relates to attitudes to digital technologies’ ability to deliver more accessible, effective, affordable, convenient, private, and user-friendly counselling services, as well as the need for control to protect users’ safety. Factor 2 (Attitudes towards the effectiveness of online therapies, and trust in AI) relates to respondents’ evaluations of the relative merit of online versus traditional face-to-face therapies, the perception of the inevitability of AI technologies, and level of trust in AI. Finally, Factor 3 (Perception of the importance of data protection and human endorsement)– relates to perceptions of the importance for mental health apps to have good data protection, to be endorsed by other students and professionals, and to made locally.

Two of the survey items did not load onto any of the above factors and we retained these as individual items in the final regression models. They were ‘Perception of the importance of data protection and privacy’, and ‘Perception that online therapy programs are less effective than conventional face-to-face therapy’. The factor loadings for all factors are shown in Table 4.

**Table 4.** Factor loadings.

Rotated Factor Pattern (Standardised Regression Coefficients)			
	Factor1	Factor2	Factor3
Digital technologies could help to deliver more accessible student counselling services	0,82769	.	.
Digital technologies could help to deliver more effective student counselling services	0,76020	.	.
Digital technologies could help to deliver more affordable student counselling services	0,83949	.	.
Digital technologies could help to deliver more convenient student counselling services	0,86365	.	.
Digital technologies could help to deliver more private and more anonymous student counselling services	0,68044	.	.
Digital technologies could help to deliver more user-friendly student counselling services	0,72735	.	.
The use of digital technologies to provide mental health services needs to be controlled by legislation and regulations to protect users	0,65075	.	.
I believe superhuman AI (i.e., artificial intelligence that is even smarter than people) will be developed within the next 1 years	.	0,53578	.
If I had to have a blood test, I would prefer to have the blood drawn by an AI robot, rather than a nurse/doctor	.	0,63348	.
Data protection and privacy	.	.	0,62675
Recommendations of other students	.	.	0,58673
Recommendations by a mental health professional	.	.	0,70450
Apps developed specifically for students	.	.	0,64975
Apps developed by South Africans	.	.	0,56831
Knowing that the app was approved by the health professions council or the government department of health	.	.	0,72080
Online therapy will eventually replace face-to-face therapy	.	0,69126	.
Online therapy is more convenient than face-to-face therapy	.	0,55465	.
Online therapies are more private because you don't have to go to the counselling centre to get help	.	0,64606	.
Trust in a therapist can be just as easily built on the Internet as in conventional face-to-face psychotherapy	.	0,70158	.
I am concerned about data security and privacy when I use apps and other digital technologies	.	.	.
Online therapy programs are less effective than conventional face-to-face therapy	.	.	.

*Values less than 0.4 are not reported.

### Regression analysis of factors associated with intention to use digital mental health solutions

The results of the multivariate ordinal regression analysis of factors associated with the intention to use various digital mental health solutions, controlling for demographic variables and controlling for intention to use traditional therapies, are shown in Table 5.

**Table 5.** Factors associated with students’ intention to use digital mental health solutions (online therapy, internet searches, apps, and chatbots).

		Intention to use online therapy			Intention to use online searches			Intention to use apps			Intention to use chatbots		
		OR	95% CI		OR	95% CI		OR	95% CI		OR	95% CI	
Gender	Male	0.93	0.87	1.01	0.98	0.91	1.06	0.85 *	**0**.**79**	**0**.**92**	1.15 *	**1**.**07**	**1**.**25**
	Gender non-conforming	1.52 *	**1**.**01**	**2**.**31**	0.97	0.66	1.45	0.95	0.64	1.41	0.73	0.46	1.14
Population group	Black-African	1.06	0.94	1.2	1.04	0.92	1.17	1.32 *	**1**.**17**	**1**.**49**	2.18 *	**1**.**89**	**2**.**51**
	Coloured	1.00	0.83	1.19	1.19	0.99	1.42	1.21	0.99	1.43	1.35 *	**1**.**10**	**1**.**65**
	Asian or Indian	0.71 *	**0**.**56**	**0**.**86**	1.26 *	**1**.**02**	**1**.**57**	1.18	0.95	1.46	1.62 *	**1**.**28**	**2**.**05**
	Other	1.17	0.86	1.59	1.08	0.81	1.46	1.27	0.94	1.72	1.52 *	**1**.**09**	**2**.**12**
Student status	Undergraduate student	0.84 *	**0**.**76**	**0**.**92**	0.98	0.89	1.07	0.99	0.90	1.08	1.16 *	**1**.**05**	**1**.**28**
Willingness to use traditional therapies		2.64 *	**2**.**54**	**2**.**75**	1.64 *	**1**.**59**	**1**.**70**	1.78 *	**1**.**72**	**1**.**85**	1.46 *	**1**.**41**	**1**.**52**
Factor 1: Attitudes towards digital technologies’ utility to improve student counselling services, provided they are safe		2.17 *	**1**.**98**	**2**.**37**	3.18 *	**2**.**91**	**3**.**48**	3.18 *	**2**.**91**	**3**.**48**	2.59 *	**2**.**36**	**2**.**85**
Factor 2: Attitudes towards the effectiveness of online therapies, and trust in AI		1.95 *	**1**.**82**	**2**.**10**	1.24 *	**1**.**15**	**1**.**33**	1.75 *	**1**.**63**	**1**.**88**	2.56 *	**2**.**38**	**2**.**76**
Factor 3: Perception of the importance of data protection and human endorsement		1.31 *	**1**.**19**	**1**.**42**	1.23 *	**1**.**14**	**1**.**34**	1.26 *	**1**.**15**	**1**.**37**	1.14 *	**1**.**04**	**1**.**24**
Perception of the importance of data protection and privacy		1.03	0.98	1.08	0.94 *	**0**.**91**	**0**.**99**	1.04	0.99	1.08	1.11 *	**1**.**06**	**1**.**17**
Perception that online therapy programs are less effective than conventional face-to-face therapy.		0.74 *	**0**.**70**	**0**.**77**	0.92 *	**0**.**88**	**0**.**96**	0.84 *	**0**.**80**	**0**.**87**	0.86 *	**0**.**82**	**0**.**90**
$\chi^{2}(13)$		4130.30			2166.50			2975.50			2670.40		
*p* Value		<0.0001			<0.0001			<0.0001			<0.0001		
$R^{2}$		0.327			0.187			0.246			0.227		

All models were statistically significant (*p* < 0.001), explaining between 18.7% and 32.7% of the variance in intention to use different digital solutions. The model which explained the most variance concerned intention to use online therapy ( $\chi^{2}(13)=4130.0$ , *p* = <,0001), accounting for 32.7% of the variance. The models for apps ( $\chi^{2}(13)=2975,5$ , *p* = <,0001) and chatbots ( $\chi^{2}(13)=2670,4$ , *p* = <,0001) were also significant, explaining 24.6% and 22.7% of the variance in intentions to use each, respectively. The model which explained the least variance was for internet searches ( $\chi^{2}(13)=2166.50$ , *p* = <,0001), as it accounted for only 18.7% of the variance in intention to use this solution.

Intention to use traditional therapies was significantly associated with intention to use all forms of digital therapy, although the strength of association was significantly strongly stronger for intention to use online therapy (OR: 2.64, CI = 2.54 to 2.75) and significantly least strongly associated with willingness to use chatbots (OR: 1.46, CI = 1.41 to 1.52), compared to online searchers (OR: 1.64, CI = 1.59 to 1.70) and apps (OR: 1.78, CI = 1.72 to 1.85).

Factor 1 (Attitudes towards digital technologies’ utility to improve student counselling services, provided they are safe) bore the strongest association with intention to use all four digital mental health solutions. Factor 1 was associated with a two-fold increased odds of intending to use online therapy (OR: 2.17, 95% CI = 1.98 to 2.37), which was significantly larger than the ORs observed for the factor's association with other kinds of interventions. For the other digital interventions, Factor was associated with a 3 times greater odds of intending to use internet searches (OR: 3.18, 95% CI = 2.91 to 3.48), a 3 times greater odds of intending to use apps (OR: 3.18, 95% CI = 2.91 to 3.48), and a 2.5 times greater odds of intending to use chatbots (95% CI = 2.36 to 3.85), but these ORs were not significantly different from one another.

Factor 2 (Attitudes towards the effectiveness of online therapies, and trust in AI) was significantly associated with intention to use all digital mental health solutions, although the association was significantly stronger for intention to use chatbots (OR: 2.56, 95% CI = 2.38 to 2.76) compared to intention to use online therapy (OR: 1.95, 95% CI = 1.82 to 2.10), online searches (OR: 1.24, CI = 1.15 to 1.33), or use apps (OR: 1.75, 95% CI = 1.63 to 1.88).

Factor 3 (Perception of the importance of data protection and human endorsement) was significantly associated with intention to us online therapy (OR: 1,3, CI = 1,19 to 1,42), online searches (OR: 1,23, CI = 1,14 to 1,34), apps (OR: 1,26, CI = 1,15 to 1,37), and chatbots (OR: 1,14, CI = 1.04 to 1,24), with no significant differences between these ORs indicating that the strength of the association is not significantly different across the various digital solutions.

The item ‘perception that online therapy programs are less effective than conventional face-to-face therapy‘ was not significantly associated with intention to use online therapy or intention to use apps, but was inversely associated with intention to use online searches (OR = 0.94, 95% CI = 0.90–0.99) and positively associated with intention to use chatbots (OR = 1.11, 95% CI = 1.06–1.17), although none of these ORs were very large.

The item ‘perception of the importance of data protection and privacy’ was associated with a significantly higher odds of intending to use chatbots (OR: 1.11, 95% CI = 1.06 to 1.17), and a significantly lower odds of intending to use internet searches (OR: 0.94, CI = 0.9 to 0.99).

Significant associations were observed between some demographic characteristics and intention to use the various digital mental health solutions. For gender, male respondents, relative to female students) had a significantly lower odds of intending to use apps (OR: 0.85, 95% CI = 0.79 to 0.92), but significantly higher odds of intending to use chatbots (OR:1.15, 95% CI = 1.07 to 1.25). Gender non-conforming respondents, compared to female respondents, had a significantly higher odds of intending to use online therapy (OR: 1.52, 95% CI = 1.01 to 2.31). For population group, Asian/Indian students were significantly less likely than white students to intend to use online therapies (OR: 0.7, CI = 0.56 to 0.86), but more likely to use online searches (OR: 1.26, CI = 1.02 to 1.57) and chatbots (OR: 1.62, CI = 1.28 to 2.05). Coloured students were significantly more likely than white students to use chatbots (OR: 1.35, CI = 1.10 to 1.65). Black-African students were more likely than white students to express intention to use apps (OR: 1.32, CI = 1.17 to 1.49) and chat-bots (OR: 2.18, CI = 1.89 to 2.51). Compared to post-graduate students, undergraduates were less likely to use online therapy (OR: 0.84, CI = 0.76 to 0.92) but more likely to use chat-bots (OR: 1.16, CI = 1.05 to 1.28).

## Discussion

This is the first study investigating SA university students’ attitudes towards, intention to use, and current engagement with, digital mental health solutions. Strengths of the study include the large sample drawn from four SA universities, and the insights that the data provide regarding the factors associated with students’ intention to use digital mental health solutions. In addition, the data show that a small proportion of SA university students are already using digital mental health solutions: with 12.4% having ever used an app for their mental health and 9.2% having consulted a mental health professional online. However, up to 60% of students said they intended to use a digital solution if they encountered a psychological problem in the future. This mirrors findings from other parts of the world,^33^ showing students’ general willingness to engage with novel technologies for their health and wellbeing.

Respondents in this study reported far less experience with chatbots than other digital solutions (only 4% had used a mental health chatbot). While several recent studies have showcased chatbot's potential to reduce mental health symptoms,^34,35^ and people's willingness to use chatbots,^36,37^ the fact that these are a newer technology than apps or online therapy may go some way in explaining their relatively lower use among students. Further, research has shown that many users may be reticent to utilise artificial intelligence-enabled technologies in the context of healthcare in particular, in part due to concerns about accuracy, cyber-security, and the inability of AI-led services to empathise or deal with ‘severe’ illness.^38,39^ This seems to be borne out in our data, as the factor ‘trust in AI’ had the largest relationship to the intention to use chatbots (OR: 2.81, 95% CI = 2.50 to 3.16). However, in this study, the intention to utilise a chatbot was high, with 81.1% of respondents reporting intention to use it for their mental health. This suggests that possibly familiarity, rather than attitudes, underlie low current utilisation.

Our data found that university students in SA are currently using digital solutions primarily to address depression, anxiety and stress, and to engage in mindfulness training. A 2019 systematic review suggested that internet interventions for mental health in university students had the greatest impacts on depression, anxiety and stress.^30^ As such, patterns of use among SA university students correspond to the types of digital mental health solutions with the most evidence to support them.

Our data also provides useful insights into how students in SA who use these technologies engage with them. The data show that 42.4% of students learnt about mental health apps via an internet search and 33.4% via social media, including social media advertising. This may be cause for concern, given that there has been a proliferation of mental health apps in the past decades, many of which have not been empirically tested.^31^ Relying on social media, including advertising, to identify mental health supports may not be optimal. Indeed, initiatives such as Psyberguide (https://onemindpsyberguide.org/) where experts review apps and provide user recommendations, may enable students to identify empirically supported apps. There are other examples of similar guidance initiatives such as that provided by the NHS in the UK (NHS-Mental-Health-Apps.pdf (bsuh.nhs.uk)) where the government recommends apps to potential service users. In SA, there may be opportunities for student counselling services to create platforms which enable students to make informed choices about which types of digital solutions are credible, and how to access them.

Interestingly, our data showcased a high intention to use digital mental health solutions. However, studies from other settings, including the US,^41,42^ have found mixed results in this respect. Levin, Stocke^41^ for instance, found relatively low interest in using online self-help resources among US college students. In another study from the US, Kern, Hong^33^ reported that 26.1% of respondents were open to using a mental health app. This is far lower than the present sample, in which 53.6% of respondents were open to using a mental health app. Notably, we also found that there is a group of students – 12.6% in this sample – who intend to use digital mental health solutions, but do not intend to use traditional therapies. This seems to suggest a unique group of individuals for whom digital mental health solutions will offer a novel pathway into care, that traditional services might have missed entirely.

Our findings strongly suggest that particular attitudes towards and perceptions of technology are associated with the intention to use various digital mental health solutions. Particularly, attitudes towards digital technologies’ utility to improve student counselling services, provided they are safe, are particularly important in shaping intention to use digital mental health solutions. This, in turn, suggests that it may be possible to increase students willingness to use digital solutions through psycho-educational interventions designed to change these specific undergirding attitudes. For example, in this study, students who are more likely to use all of the digital interventions we investigated think that technology is a more accessible way to deliver services, suggesting that maybe stressing the accessibility of these technologies may promote the uptake of these technologies. Likewise, the intention to use apps was negatively associated with the perception that online therapy programs are less effective than conventional face-to-face therapy, suggesting that attitude change interventions around the effectiveness of online therapies could have knock-on effects on students’ willingness to use apps. Similarly, intention to use chatbots was associated with the lower perceived importance of the need for regulation and security of digital mental health solutions, suggesting that individuals who are willing to use chatbots may have a higher level of comfort with and confidence in, digital technologies, or, alternatively, low perceived need for regulation. Both of these findings show clear entry points for intervention to increase students’ engagement with digital mental health solutions. This may be important, because, as noted in the introduction, in many LMICs, resources to implement mental health programming are scarce, and digital interventions are increasingly used.^43^ If available resources are being used for digital solutions, students need to be sensitised and equipped to engage with them.

There were also some associations between demographic characteristics of respondents, and intentions to engage with different digital mental health solutions. We explored these associations largely because inequalities in access to digital technologies (the ‘digital divide’) are often along the lines of historical disadvantage, and in SA, historical disadvantage is mapped along population group lines. So, for equity reasons, these differences are important to note. For instance, we found that gender non-conforming respondents, relative to male and female respondents, had a significantly higher odds of intending to use online therapy (OR: 1.52, 95% CI = 1.01 to 2.31).

Black-African students were more likely than white students to express intention to use apps (OR: 1.32, CI = 1.17 to 1.49) and chat-bots (OR: 2.18, CI = 1.89 to 2.51), than white students. Given that SA evidence suggests both gender non-conforming and Black-African students are at risk of being missed by mental health services on university campuses,^11^ it will be important to consider what these findings means. Qualitative work to understand the complex determinants of variation along lines of gender and population group is needed.

Finally, an incidental but notable finding of this research is that our study is the first in SA of which we are aware to show that a higher frequency of social media use is associated with poorer self-reported mental health status among students. Recent high-quality studies^44^ suggest that for most people social media use and screen time have minimal impact on mental health. Most studies^45^ focus narrowly on adults, have only investigated correlations (not causation), and have failed to identify any consistent or large positive effects. Although large scale rigorous studies on adolescents have found small positive associations between poor mental health and duration of daily technology use,^46^ they do not distinguish cause from effect and thus cannot explain if poor mental health causes adolescents to spend more time using digital technologies, or vice versa. Nonetheless, these findings do raise the question that technology may have some negative impacts on young people's mental health. Yet, interest and investment in digital mental health solutions continues to grow. The possibility that digital mental health solutions may be contraindicated for some young people needs to be explored as it is plausible that those who are high social media utilisers and whose utilisation negatively impacts their mental health, may be at risk of iatrogenic effects from digital mental health interventions.

## Limitations

We relied on a self-selected sample of students from only 3 of the country 26 universities, which affects the generalisability of our findings.

## Conclusion

One of the tensions implicit in digital mental health is that the pervasiveness of digital technologies in young people's lives creates challenges, but also offers unprecedented opportunities to intervene using digital mental health solutions. This study suggests that a small proportion of SA university students are currently engaging with digital mental health online therapy, mental health apps, and even chatbots. Furthermore, their willingness to do so appears high (53.6% - 68.5% of respondents intended to use one or more digital mental health solutions). This suggests an unprecedented opportunity to reach new service users through technology. Finally, our study shows that respondents who perceive digital mental health solutions as improving mental healthcare access, effectiveness, affordability, convenience, privacy and user friendliness, have greater intentions to engage with digital mental health solutions. This points to possible avenues for attitude change interventions to increase utilisation.

## Supplemental Material

sj-docx-1-dhj-10.1177_20552076231216559 - Supplemental material for Intention to use digital mental health solutions: A cross-sectional survey of university students attitudes and perceptions toward online therapy, mental health apps, and chatbots Click here for additional data file. Supplemental material, sj-docx-1-dhj-10.1177_20552076231216559 for Intention to use digital mental health solutions: A cross-sectional survey of university students attitudes and perceptions toward online therapy, mental health apps, and chatbots by Elton Fayiah Gbollie, Jason Bantjes, Lucy Jarvis, Sonja Swandevelder, Jean du Plessis, Richard Shadwell, Charl Davids, Rone Gerber, Nuhaa Holland and Xanthe Hunt in DIGITAL HEALTH
