# Supplementary material for: Intention to use digital mental health solutions: A cross-sectional survey of university students attitudes and perceptions toward online therapy, mental health apps, and chatbots
Source: Digit Health. 2023 Nov 30;9:20552076231216559. doi: 10.1177/20552076231216559 (PMC10693229; doi:10.1177/20552076231216559)
Supplement: sj-docx-1-dhj-10.1177_20552076231216559 - Supplemental material for Intention to use digital mental health solutions: A cross-sectional survey of university students attitudes and perceptions toward online therapy, mental health apps, and chatbots [file sj-docx-1-dhj-10.1177_20552076231216559.docx]

| **Table x:** Mental health status and treatment history among students who are willing to use digital mental health technologies | | | | | |
| --- | --- | --- | --- | --- | --- |
|  | Self-reported mental health status | | | | Received treatment in the past |
|  | Poor | Fair | Good | Excellent |  |
| Willingness to use traditional therapy (n=10889) | 917 | 2898 | 4007 | 3067 | 2625 |
|  | 8.4% | 26.6% | 36.8% | 28.2% | 24.1% |
| Willingness to use online therapy (n=9167) | 798 | 2367 | 3307 | 2695 | 2133 |
|  | 8.7% | 25.8% | 36.1% | 29.4% | 23.3% |
| Willingness to use Internet searches for psychoeducational materials (n=10457) | 842 | 2735 | 3837 | 3043 | 2318 |
|  | 8.1% | 26.2% | 36.7% | 29.1% | 22.2% |
| Willingness to use aps (n=8147) | 650 | 2032 | 2998 | 2467 | 1630 |
|  | 8.0% | 24.9% | 36.8% | 30.3% | 20.0% |
| Willingness to use chatbots (n=5197) | 376 | 1109 | 1846 | 1866 | 794 |
|  | 7.2% | 21.3% | 35.5% | 35.9% | 15.3% |
| Willingness to use any digital mental health technology (12731) | 1112 | 3398 | 4648 | 3573 | 2914 |
|  | 8.7% | 26.7% | 36.5% | 28.1% | 22.9% |

| **Table x:** Mental health status and treatment history among students who previously use various digital mental health technologies | | | | | |
| --- | --- | --- | --- | --- | --- |
|  | Self-reported mental health status | | | | Received treatment in the past |
|  | Poor | Fair | Good | Excellent |  |
| Previously used online therapy (n=1626) | 292 | 645 | 474 | 215 | 1085 |
|  | 18.0% | 39.7% | 29.2% | 13.2% | 66.8% |
| Previously used spps (n=1944) | 296 | 790 | 589 | 269 | 875 |
|  | 15.2% | 40.6% | 30.3% | 13.8% | 45.0% |
| Previously used chatbots (n=717) | 94 | 231 | 236 | 156 | 287 |
|  | 13.1% | 32.2% | 32.9% | 21.8% | 40.1% |
| Previously used any of the above mental health technologies (n=3349) | 528 | 1297 | 1046 | 478 | 1624 |
|  | 15.8% | 38.7% | 31.2% | 14.3% | 48.5% |
| Note: We did not ask about previous use of online searches for psychoeducational mental health materials | | | | | |
